# Why and how a university in Rwanda is training its medical students in one health

**DOI:** 10.1038/s43856-022-00218-0

**Published:** 2022-11-27

**Authors:** Ursin Bayisenge, Kelsey Ripp, Abebe Bekele, Phaedra Henley

**Affiliations:** 1 University of Global Health Equity, Butaro, Rwanda; 2 Alaska Native Tribal Health Consortium, Anchorage, AK USA

**Keywords:** Preventive medicine, Epidemiology, Physical examination

## Abstract

Bayisenge et al. describe teaching One Health approaches to medical students at the University of Global Health Equity in Rwanda. Wider implementation of this approach should enable a better response to the health challenges of our changing planet.

## Teaser

The One Health approach considers the contribution of animals and the environment to health. Here we describe how the University of Global Health Equity in Rwanda is integrating One Health into its medical curriculum to train the next generation of doctors to respond to the health challenges of our changing planet.

The One Health approach recognizes the inextricable links between the health of humans, animals, and their shared environment. It differs from other approaches to health as it considers the integrative effort of multiple disciplines and sectors working locally, nationally, and globally to achieve evidence-based and community-placed solutions to complex health challenges such as zoonotic diseases like COVID-19, climate change, and food security. It is an interdisciplinary approach that brings different professionals together to think about and act on related health threats^1^. While One Health is a recently developed term, the understanding that the health of humans, animals, and the land they share is interdependent aligns with Indigenous worldviews that can be traced back over tens of thousands of years. Over 2000 years ago, the ancient Greek philosopher Hippocrates stated that human health is dependent on the environment and two centuries ago physician Rudolf Virchow stated that there is no line between human and animal health^2^. In the early 2000s, outbreaks of international concern such as severe acute respiratory syndrome (SARS) and the avian influenza H5N1 reemphasized the interconnection between human, environment, and animal health, which led major global organizations such as the World Health Organization (WHO), the Food and Agriculture Organization (FAO), the World Organization for Animal Health (WOAH), and the United Nations Environmental Program (UNEP) to embrace the One Health approach to prevent future emerging infectious diseases^1^. In late 2021, these four international organizations formalized a common definition of One Health and are working together to operationalize the approach^3^.

While these international agencies have adopted One Health as an important approach to health, implementation has been limited^4^. Such limitations are very apparent when considering how healthcare professionals are educated. Many of the global health challenges we face cross disciplines and borders, lie at the animal-environment-human interface, and require collaborative efforts to be effectively addressed. This widening responsibility for health across professions brings with it the recognition that an important transformation in attitudes and practices is required for the upcoming workforce. The training of medical students in One Health has been limited compared to veterinary and environmental health academic training^5,6^. Medical students from Canada, United States of America, United Kingdom, Malaysia, and Ireland have developed a planetary health report card to assess the level of inclusion of the impact of global environmental changes on people’s health in medical school curricula^7^. However, this tool does not specify the inextricable link between humans and animals, which is fundamental to a One Health approach.

The University of Global Health Equity (UGHE) in Rwanda is filling this gap through practical One Health training for all students, including those studying medicine. At UGHE, students studying medicine learn about the One Health approach during both pre-clinical and clinical training. This approach to medical education will encourage a holistic mindset and provide a wider set of practical tools to address human health threats. To our knowledge, UGHE is one of the first academic institutions in the world to invest in the integration of the One Health approach in the entire undergraduate medical program.

Training in One Health will influence the way future medical doctors conduct patient care in practice by considering environmental and animal contact while obtaining a medical history, performing a physical exam, determining a diagnosis and management plan, providing patient education, and conducting research^6^. The added value of One Health-focused clinical skills includes enhancing diagnosis of health conditions influenced by the environment, strengthening the capacity to detect unexpected diseases and new threats, contributing to a fuller understanding of a patient’s illness and the varied components needed for treatment. This should enable clinicians to predict and adapt to anticipated changes in medical care as our planet changes.

At UGHE, One Health faculty, biomedical and medical professionals, public health and environmental health specialists, and veterinary specialists work together to identify and teach components of a clinically-oriented curriculum using a multidisciplinary perspective to enhance learning and practice. In preclinical years, medical students have a weeklong introductory course on One Health. After this, there are dedicated One Health sessions in modules, from the Cardiovascular System to Infectious Disease, that focus on a One Health approach to module-specific topics, such as air pollution and cardiovascular health. There are additional sessions in which One Health teaching points are inserted into existing sessions. This allows students to systematically and consistently practice applying a One Health perspective throughout their preclinical teaching and also enables the inclusion of a One Health curricular theme without overwhelming an already busy medical curriculum. In the near term, One Health will also be integrated into the innovative community-based education component of UGHE’s medical degree program. This will train students to see the importance of looking beyond the biomedical aspects of health and motivate them to work collaboratively with communities and other professionals to find sustainable and comprehensive solutions for their patients. Furthermore, UGHE’s undergraduate medical degree program is a dual degree with a Master of Science in Global Health Delivery, allowing the students to complete additional One Health-related modules such as public health policy and implementation.

During the clinical training years, students practice skill-based One Health competencies, such as taking a One Health history (Box 1), and then apply this information to clinical scenarios both at the bedside and in the classroom. During the Introduction to the Practice of Medicine course, students are taught how to take a One Health history alongside a comprehensive patient history. This enables the One Health approach to be implemented from the very first days of clinical training. To further facilitate the integration of One Health into clinical rotations, a One Health clinical guide for students and facilitators has been developed to demonstrate how the approach can be used within clinical practice while at the same time ensuring that the content does not deviate from or overburden the curriculum. The clinical guide is a handbook that helps students to ask relevant questions about animal interactions and environmental exposures, probe into clues for sentinel diseases affecting humans and animals and discover key aspects of management for key climate-change affected conditions such as heat stress or air pollution. It builds on teaching in preclinical modules and demonstrates how to apply this knowledge to patient care. Teaching materials, such as examples of dialogs that could be had with patients and clinical cases designed for case-based collaborative learning, are used to demonstrate the feasibility of a One Health approach inside and outside clinical settings. The long-term vision is to build a One Health clinic where medical students will spend dedicated time with veterinary students to learn from one another about comparative medicine in a multidisciplinary environment.

## Box 1

Example of the benefits of taking a One Health history.

One case study from the preclinical Integumentary System (Skin) module covers a patient with bilateral limb swelling. Recommended questions to enable a One Health history to be taken include “Does she live in an area with volcanoes nearby? How long has she lived there? Does she have other family members with lower limb swelling?” By taking a One Health history, it is possible to determine that podoconiosis is the cause of the patient’s bilateral limb swelling. Podoconiosis occurs in farmers who do not wear shoes and are in long-term contact with irritant red clay soil of volcanic origins. The patient can also be educated about how provision of protective footwear whilst undergoing farming activities could prevent it in the future^10^.

The One Health approach taught through this case study can also be practiced by students in their clinical years, for example, in a clinical case presented at morning report.

The integration of One Health in medical training presents several challenges. Existing academic regulations in medical schools at national and global levels assume a discipline-based education and do not provide scope for interdisciplinary education^6^. An already full curriculum does not encourage medical schools to consider additional material including those on One Health. Promotion and demonstration of the success of the integration approach at UGHE needs to be disseminated. Various teaching materials and faculty support are needed to facilitate the integration of One Health concepts, which could be an additional financial and time burden. At UGHE, the Center for One Health delivers trainings to build capacity of our faculty to ensure that One Health is effectively integrated in all relevant medical modules. Training materials and clinical guides are being developed to guide One Health integration both at the institution and more broadly.

Training in One Health at all biomedical and medical higher learning institutions would enable the world to be better prepared for, and responsive to, emerging health threats, such as future pandemics and the adverse effects of climate change on population health outcomes, including infant mortality and life expectancy^8^. The power of the One Health approach in biomedical practice is indisputable and its success lies in the implementation of quality interdisciplinary education that will shape the attitude and practice of future health leaders^9^.
